# Anesthesia Practices for Preterm Infants: A Survey in the Nordic Countries and Review of the Literature

**DOI:** 10.1111/aas.70186

**Published:** 2026-02-12

**Authors:** Jaap van der Heijden, Peter Frykholm

**Affiliations:** ^1^ Department of Surgical Sciences, Anesthesiology and Intensive Care Uppsala University Hospital Uppsala Sweden

**Keywords:** induction, maintenance, pediatric anesthesia, perioperative, preterm infants, vasopressor

## Abstract

**Background:**

Preterm infants are at increased risk of perioperative mortality and are among the most vulnerable patients anesthesiologists can encounter. The literature on anesthesia practice in preterm infants is limited, mainly focusing on their physiological immaturity and related pharmacodynamics and pharmacokinetics.

**Aims:**

Our aim was to gather comprehensive data on the current practice of anesthesia management in preterm infants and, when available, to compare these findings with existing literature or guidelines.

**Methods:**

A cross‐sectional study was conducted using a structured survey distributed to pediatric anesthesiologists across the Nordic countries between June and November 2024. The survey assessed various aspects of anesthesia practice in preterm patients, including agent dosing for induction and maintenance, perioperative monitoring techniques, and respiratory and circulatory management strategies.

**Results:**

A total of 57 surveys were collected from 14 hospitals, with an estimated response rate of 30.4%. For induction, 15 different combinations were reported, with the most common being fentanyl and sodium thiopental (31.6%). For maintenance, 13 combinations were identified, primarily fentanyl and sevoflurane (47.4%). Anesthetic doses exhibited considerable variability, with up to a 20‐fold difference between the lowest and highest doses. Rocuronium and atracurium were used almost equally as neuromuscular blocking agents, although significant variation in dosing intervals was noted. Targeted mean SpO_2_ levels ranged from 90% to 96%, and EtCO_2_ levels from 4.5 to 6.5 kPa, with some outliers. Approximately 86% of respondents used gestational age in weeks as the minimum acceptable mean arterial pressure, with noradrenaline being the first‐choice vasopressor (59.6%). Notable differences were observed in transfusion thresholds, fluid resuscitation strategies, and monitoring practices among respondents.

**Conclusion:**

The substantial heterogeneity in anesthesia practice highlighted the need for high‐quality research leading to evidence‐based protocols and broader consensus to improve safety and quality of care for this vulnerable population.

This survey reports on the breadth of practice details for anesthesia management when preterm infants require surgery, from centers in the 5 Nordic countries.

## 1 Introduction

Preterm infants are among the most vulnerable patients encountered by pediatric anesthesiologists. The postoperative thirty‐day mortality for neonates is 3.7%–10.9% and the risk increases with lower postmenstrual age [1, 2, 3]. This high mortality rate is primarily related to the indication for surgery, complications, severe comorbidities, and prolonged post‐operative ventilation rather than anesthesia. The available literature on anesthesia practice in preterm infants is limited and primarily emphasizes the physiological differences, that is, organ immaturity including the airway and the gastrointestinal, respiratory, cardiovascular, and neurological systems. Additionally, pharmacodynamic and pharmacokinetic differences specific to this vulnerable population have been stressed [4, 5].

A European survey recently described a broad overview of anesthesia practice for preterm infants across Europe [6]. However, information about several important aspects remains incomplete including dosing of induction agents and maintenance of anesthesia, the use of neuromuscular blocking agents, and intervention thresholds for oxygen saturation (SpO_2_), carbon dioxide (CO_2_), blood pressure and transfusion.

The aim of the study was to address this gap in knowledge by sending a survey to pediatric anesthesiologists in the Nordic countries to collect more detailed information about the current management of anesthesia in preterm infants, and to compare the results with current literature or guidelines if available.

## 2 Methods

### 2.1 Study Design and Data Collection

This was a cross‐sectional survey‐based study. Tertiary centers in the Nordic countries (Denmark, Finland, Iceland, Norway, and Sweden) were eligible if they were equipped with a neonatal intensive care unit (NICU) as well as a dedicated pediatric anesthesia service. Approval by the Swedish Ethical Review Authority was not deemed necessary, given that no collection or handling of sensitive or patient‐related data was part of the study. This study was reported according to the Checklist for Reporting of Survey Studies (CROSS) [7].

The web‐based software platform Research Electronic Data Capture (REDCap, Uppsala University) was used for survey design and data collection [8, 9]. The survey consisted of 32 questions and included several aspects of anesthesia in preterm infants such as type of agents used for induction and maintenance, perioperative monitoring routines, and ventilatory and circulatory strategies. The complete survey is available in the e‐supplement (Data S1). When appropriate, specification of dosage, interval, or lowest/highest threshold before intervention were added as follows: follow‐up questions. To include all possible answers and minimize the risk of missing data, the option ‘other’ was given for selected questions with free‐text options.

The survey was first distributed in June 2024 with two reminders sent in August and October. The head of the department of each of the 18 selected hospitals was requested to forward the survey to all anesthesiologists who had performed anesthesia on preterm infants during the preceding 12 months. They were further encouraged to reply how many anesthesiologists received the survey. The survey participants were requested to answer the questions according to a specific scenario (case description below) that was presented at the start of the survey. This case concerns necrotizing enterocolitis, a relatively common indication for surgery in preterm infants. The survey was closed in November.

### 2.2 Statistical Analysis

Those who were not authorized to perform anesthesia on preterm infants independently were excluded to avoid redundant (double entry) input from teaching cases and to minimize the risk of including answers from less experienced anesthesiologists. Data was presented as median and IQR or range when appropriate. The possible association between combinations of agents used and hospital or respondents' experience (stratified into ≤ 10 years and > 10 years) was examined using Fisher's exact test with Monte Carlo simulation (*B* = 20,000). Data processing and boxplot generation were performed using Excel (version 16.77.1). Fisher's exact test and UpSet plots and heatmaps were done in RStudio (version 2024.09.0 + 375).

**Table:** 

Case description: specific scenario presented at start of the survey	
S	Acute necrotizing enterocolitis
B	Preterm, born week 24, postmenstrual age week 27, mild bronchopulmonary dysplasia
A	A – normal facial anatomy, previously intubated and extubated without problems. B – intubated, FiO_2_ 45%, mechanically ventilated with peak pressure 24, PEEP 7, transcutaneous CO_2_ 6. C – adequate blood pressure and heart rate without vasopressor support, electrolytes within normal range, peripherally warm and perfused with capillary refill < 2 s, Hb 14 g dL^1^ D – no signs of syndrome or neurological defects, signs of bowel obstruction
R	R – acute laparotomy

## 3 Results

### 3.1 Demographics

Eighteen hospitals were approached and a total of 63 respondents from 14 sites (77.8%) completed the survey. Six surveys were excluded for further analysis as the respondents were not authorized to perform anesthesia on preterm infants independently. Half of the hospitals reported how many anesthesiologists received the survey (*n* = 112) and 34 of these completed the survey resulting in a response rate of 30.4% for these nine hospitals. Of the 57 respondents, 20 were female (35.1%) and 27 (47.3%) of the respondents were based in Sweden. Detailed information about the demographics, work experience and caseload are presented in Table 1.

**TABLE 1:** Demographics, work experience and workload.

Demographics			
Age (years)		51	(45–57)
Gender (*n*)	Female	20	35.1%
	Male	37	64.9%
Country (*n*)	Denmark	13	22.8%
	Finland	10	17.5%
	Iceland	1	1.8%
	Norway	6	10.5%
	Sweden	27	47.3%
Experience/Workload			
Experience after specialist training (years)		15	(10–20)
Experience in pediatric anesthesia (years)		12	7–15
Number of cases managed in 12 months		5	4–12
NICU involved perioperative (%)		3	0–10
Anesthesia during office hours (%)			
Single anesthesiologist		43	75.4%
Two or more		14	24.6%
Anesthesia after office hours (%)			
Single anesthesiologist		49	86.0%
Two or more		8	14.0%

*Note:* Data presented as absolute number of replies (%) or median (IQR).

### 3.2 Induction Agents

All but two (96.5%) respondents used fentanyl during induction of anesthesia with a 10‐fold difference between the lowest and highest dosing used. Remifentanil and ketamine were used by two (3.5%) and 27 (47.4%) of the respondents respectively. Propofol was used by 15 (26.3%) respondents with 5 mg kg^−1^ as the highest induction dose reported. Sodium thiopental was used by 23 (40.4%) respondents. Other agents used were sevoflurane, lorazepam, and midazolam. All but three respondents used a neuromuscular blocking agent (NMBA) at induction and rocuronium (*n* = 25, 43.9%) and atracurium (*n* = 22, 38.6%) were the most frequently used. Atropine was used by 15 (26.3%) respondents. For detailed information about dosing and range see Table 2.

**TABLE 2:** Induction agents.

	Count	% of total	Median	Min	Max
Fentanyl (mcg kg^−1^)	55	96.5	2	1	10
Remifentanil (mcg kg^−1^)	2	3.5	0.4	0.3	0.5
Ketamine (mg kg^−1^)	27	47.4	2	0.5	4
Propofol (mg kg^−1^)	15	26.3	2	1	5
Sodium thiopental (mg kg^−1^)	23	40.4	3	2	7
Sevoflurane (ETAC %)	2	3.5	2.25	1.5	3
Lorazepam (mg kg^−1^)	1	1.8	0.1	N/A	N/A
Midazolam (mg kg^−1^)	3	5.3	0.1	0.1	0.2
Rocuronium (mg kg^−1^)	25	43.9	1	0.4	1.5
Atracurium (mg kg^−1^)	22	38.6	0.5	0.5	1
Cis‐atracurium (mg kg^−1^)	5	8.8	0.15	0.1	0.2
Succinylcholine (mg kg^−1^)	2	3.5	1.75	1.5	2
Atropine (mcg kg^−1^)	15	26.3	10	2	20
Glycopyrrolate (mcg kg^−1^)	2	3.5	5	5	5

**FIGURE 1:**
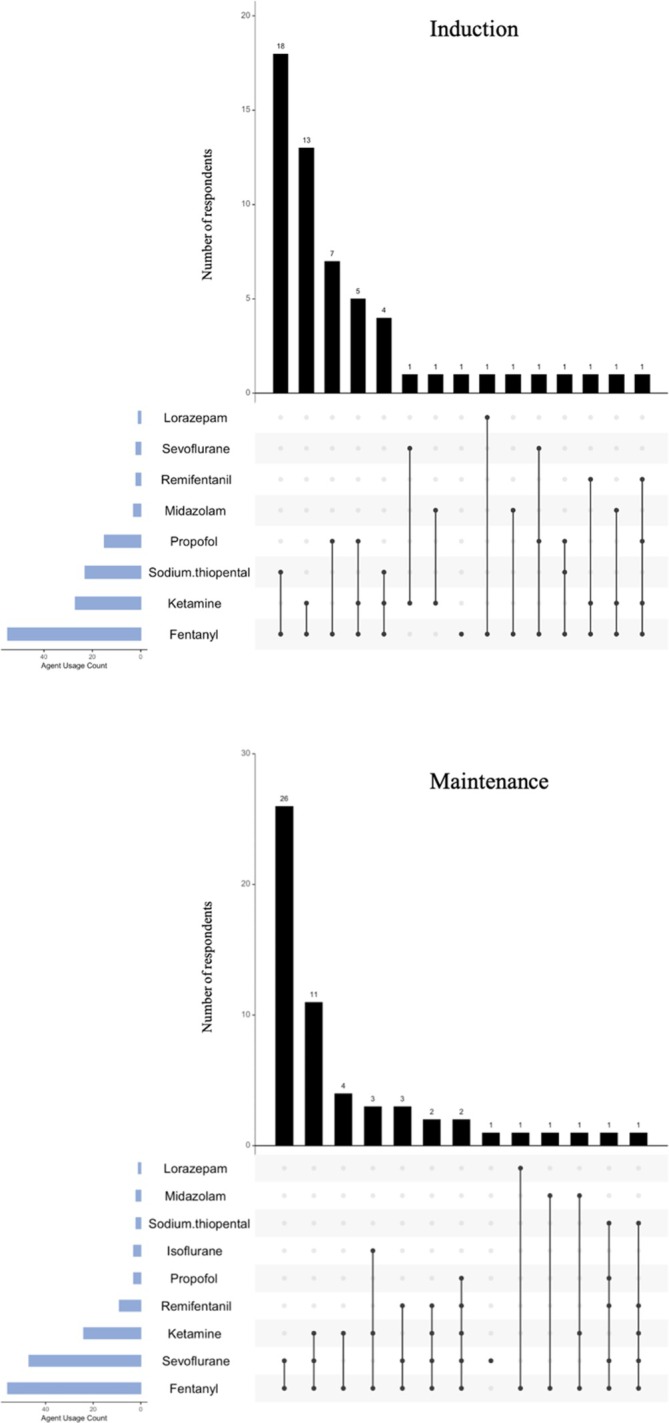
UpSet plot for anesthetic agents used for induction and maintenance of anesthesia. The horizontal bars on the left indicate the frequency with which each individual agent was used across all respondents (*n* = 57). The vertical bars at the top display the frequency of specific agent combinations. Each combination is represented by a series of connected dots.

A total of 15 combinations of pharmacological agents were identified for induction of anesthesia (Figure 1). The most common combination was fentanyl and sodium thiopental (*n* = 18, 31.6%) followed by fentanyl and ketamine (*n* = 13, 22.8%). Other common combinations were fentanyl and propofol (*n* = 7, 12.3%), fentanyl, ketamine and propofol (*n* = 5, 8.8%), and fentanyl, ketamine and sodium thiopental (*n* = 4, 7.0%). Ten combinations were used by individual respondents only. No association was found between the experience level of the respondent (≤ 10 years or > 10 years experience in pediatric anesthesia, respectively) and the use of a certain combination of agents (*p* = 0.456). However, the hospital in which an individual respondent was working was associated with the specific combination of agents they used (*p* < 0.001) (Figure S1).

### 3.3 Maintenance Agents

All but one (98.2%) respondent used fentanyl as maintenance agent with a 20‐fold difference between lowest and highest dosing used. Remifentanil infusion was used by nine (15.8%) with a 10‐fold difference between lowest and highest dose. Inhalational anesthesia was used by 50 (87.7%) respondents with a 10‐fold difference in the lowest and highest end tidal anesthesia concentration (ETAC) for sevoflurane. Ketamine bolus and infusion were used by 21 (36.8%) and 17 (29.8%) respondents with a 6‐fold and 25‐fold difference respectively between the lowest and highest reported doses (Table 3).

**TABLE 3:** Maintenance anesthetic agents.

	Count	% of total	Median	Min	Max
Fentanyl total	56	98.2			
Injection (mcg kg^−1^)	55	96.5	2	1	20
Infusion (mcg kg h^−1^)	1	1.8	2	N/A	N/A
Remifentanil (mcg kg min^−1^)	9	15.8	0.3	0.05	0.5
Ketamine total	23	40.4			
Injection (mg kg^−1^)	21	36.8	1	0.5	3
Infusion (mg kg h^−1^)	17	29.8	2	0.2	5
Propofol (mg kg h^−1^)	3	5.3	10	2	15
Sevoflurane (ETAC %)	47	82.5	1.5	0.3	3.5
Isoflurane (ETAC %)	3	5.3	0.8	0.5	0.8
Sodium thiopental (mg kg^−1^)	2	3.5	3	2.5	3.5
Lorazepam (mg kg^−1^)	1	1.8	0.1	N/A	N/A
Midazolam (mg kg^−1^)	2	3.5	0.1	0.1	0.1

*Note:* Agents stated as ‘per kg’ are administered as bolus while agents mentioned as ‘kg h^−1^’ are administered as infusion.

A total of 13 combinations for maintenance of anesthesia were identified (Figure 1). Most respondents (98.2%) used fentanyl and almost half used it exclusively together with sevoflurane (*n* = 26, 45.6%). Fentanyl was used by six (10.5%) respondents with another single agent. The combination of fentanyl, sevoflurane and ketamine was used by 11 (19.3%) respondents while the combination fentanyl, ketamine and isoflurane was used by three (5.3%). The combination of fentanyl, sevoflurane and remifentanil was used by three (5.3%) respondents and six (10.5%) used a combination of at least four different agents. One respondent selected sevoflurane as a single anesthetic. The hospital in which the respondent was working but not the experience level (≤ 10‐years or > 10‐years) was associated (*p* < 0.001 and *p* = 0.320 respectively) with a specific combination of agents used (Figure S2).

### 3.4 Neuromuscular Blocking Agents (NMBA)

For maintenance, rocuronium was the most commonly used NMBA, used by 24 (42.1%) respondents, followed by atracurium (*n* = 23, 40.4%) and cis‐atracurium (*n* = 9) (Table 4). The top‐up dose intervals ranged from 20 to 120 min.

**TABLE 4:** Maintenance NMBA and interval.

	Count	% of total	Median	Min	Max
Rocuronium (mg kg^−1^)	24	42.1	0.7	0.45	1.5
Interval (min)			60	30	120
Atracurium (mg kg^−1^)	23	40.4	0.5	0.25	2
Interval (min)			55	20	120
Cis‐atracurium (mg kg^−1^)	9	15.8	0.15	0.1	0.2
Interval (min)			45	30	60
Pancuronium (mg kg^−1^)	1	1.8	0.1	NA	NA
Interval (min)			ND	NA	NA

### 3.5 Ventilation

**FIGURE 2:**
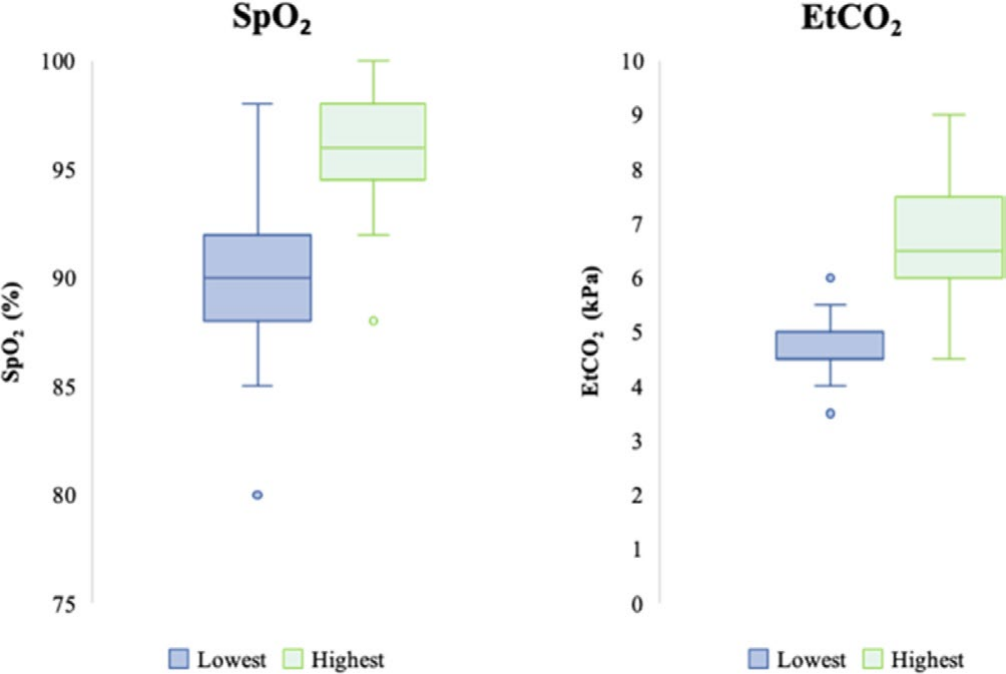
The lowest and highest acceptable values for SpO_2_ (left) and end‐tidal CO_2_ (right) to trigger intervention.

The lowest and highest SpO_2_ that the respondents accepted without intervening was 90% (IQR 88%–92%) and 96% (IQR 95%–98%, Figure 2), respectively. The corresponding range for EtCO_2_ was 4.5 kPa (IQR 4.5–5 kPa) and 6.5 kPa (IQR 6–7.25 kPa, Figure 2). The median reported tidal volume was 6 mL kg^−1^ (range 4–12 mL kg^−1^, IQR 6–7 mL kg^−1^). Pressure‐controlled ventilation was used by 49 respondents, pressure‐controlled volume guaranteed by 7 and volume controlled by one respondent (86%, 12.3%, and 1.7% respectively).

### 3.6 Circulation

All respondents used mean arterial pressure (MAP) rather than systolic or diastolic blood pressure to trigger interventions. Most (*n* = 49, 86%) respondents used the gestational age (GA) as a threshold for lowest acceptable MAP (GA in weeks = minimal accepted MAP in mmHg) while one respondent added 3 mmHg for the GA age, 2 respondents used a fixed MAP and 5 respondents used a combination of several clinical features (see Table S1). For the fictive case (postmenstrual age 27), the median hypotension threshold for intervention was 30 mmHg (IQR 27–30) and the transfusion trigger was 10 g dL^−1^ (IQR 10–11). Most respondents (*n* = 40, 70.1%) started vasopressor treatment strictly based on MAP values, eight (14.0%) used it routinely throughout the procedure, 5 (8.8%) rarely started treatment with vasopressors and four respondents used a combination of several clinical parameters.

In a euvolemic, hypotensive preterm infant, noradrenaline was the first choice of vasopressor (*n* = 34, 59.6%) followed by dopamine (17.5%), adrenaline (8.8%), dobutamine (8.8%), and phenylephrine (5.2%). Calcium‐gluconate was given in the scenario by 26 (45.6%) respondents. In a clinically hypovolemic non‐bleeding preterm infant with hypotension, albumin 5% was more common (50.9%) than ringer acetate (33.3%) (Table 5).

**TABLE 5:** Choice of fluid resuscitation in hypotensive preterm infants based on non‐bleeding hypovolemia.

Fluid	Count (*n*)	%	5 mL kg^−1^	7 ml kg^−1^	10 mL kg^−1^
Albumin 5%	29	50.9	21.1	—	29.8
Normal saline 0.9%	5	8.8	3.5	—	5.3
Octaplas	1	1.8	1.8	—	—
Plasmalyte or octaplas	1	1.8	—	—	1.8
Fresh frozen plasma	2	3.5	—	—	3.5
Ringer acetate	19	33.3	5.3	1.8	26.3

### 3.7 Monitoring

**FIGURE 3:**
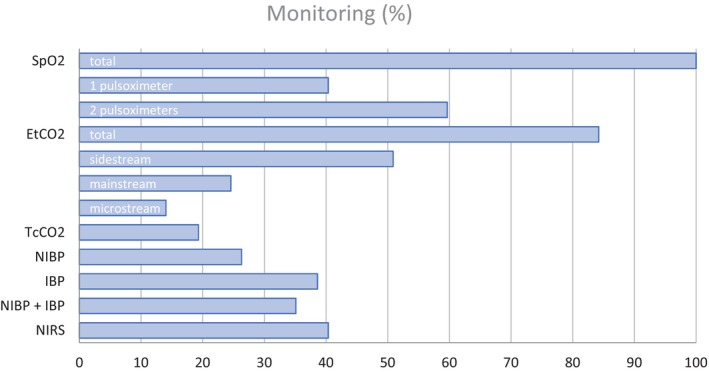
Different monitoring modalities used by respondents in % of total (*n* = 57).

Saturation was measured by all respondents with 34 (59.6%) respondents using two separate pulse oximeters. End tidal CO_2_ (EtCO_2_) monitoring was used by 48 respondents (84.2%) where several used two or more monitors simultaneously (Figure 3). Transcutaneous CO_2_ monitoring was used by 11 (19.3%) respondents. Main‐stream EtCO_2_ measurement (*n* = 24) was considered the most reliable continuous EtCO_2_ monitoring method in preterm infants followed by side‐stream (*n* = 10), transcutaneous and micro‐stream (both *n* = 8). All respondents measured blood pressure. Non‐invasive blood pressure (NIBP) and invasive blood pressure (IBP) measurements were used by 15 (26.3%) and 22 (38.6%) respectively while 20 (35.1%) used both NIBP and IBP. Near infrared spectroscopy (NIRS) was used by 23 (40.4%) of the respondents while most of them (*n* = 15) did not implement a fixed threshold value for intervention (Table S2).

## 4 Discussion

This cross‐sectional Nordic study on anesthesia management in preterm infants collected 57 surveys from 14 hospitals, achieving an estimated response rate of 30.4%. On average, anesthesiologists had performed general anesthesia five times in preterm infants in the past 12 months, highlighting the rarity of such cases. Despite the vulnerability of this patient group and the potential teaching value, most anesthesiologists managed anesthesia for preterm infants independently, both during and outside office hours. The main finding was that we found a large variation in the agents used for induction and maintenance of anesthesia, with a surprising spread between the minimal and maximal dosages administered for these agents. Furthermore, large heterogeneity of anesthesia practice was found for respiratory, circulatory, and monitoring strategies, in essence reflecting the lack of evidence to guide the clinical management of anesthesia in preterm infants.

### 4.1 Induction of Anesthesia

For preterm infants as presented in the case description, we identified 15 unique combinations for induction of anesthesia. The combination of fentanyl and sodium thiopental was the most reported combination in the Nordic countries. A substantial variability in dosing was noted, with a 10‐fold difference for fentanyl between the highest and lowest dose, a 3.5‐fold difference for sodium thiopental, an 8‐fold difference for ketamine, and a 5‐fold difference for propofol.

Nearly all respondents (96.5%) in this study used fentanyl for induction, whereas a previous European survey indicates greater variability in the choice of analgesics [6]. Anand et al. [10] demonstrated the benefits of analgesia in preterm infants undergoing surgery, with fentanyl as the preferred analgesic due to its ability to maintain hemodynamic stability, reduce stress response, and reduce pain‐induced pulmonary vascular resistance. The clearance of fentanyl in preterm infants is dependent on prenatal and postnatal age and may in part be attributed to the dynamic maturation of CYP3A7 and CYP3A4 as well as hepatic flow [11]. Notably, the half‐life of fentanyl in neonates varies considerably, ranging from 317 to 1266 min, making optimal dosing uncertain [12]. In our study, the median fentanyl dose administered was 2 mcg kg^−1^, with some respondents using doses as high as 20 mcg kg^−1^. This range may reflect that some anesthesiologists will use standard analgesic doses while others may rely on high‐dose fentanyl to allow reduced sevoflurane concentrations. A prospective study to investigate the effects of these strategies on intraoperative hemodynamic stability as well as the rate of delayed extubation in this population would be useful.

Sodium thiopental has a long history as an induction hypnotic for infants and children but its pharmacokinetic and pharmacodynamic properties in neonates are not well elucidated and studies in pre‐terms are non‐existent. A very small dose‐finding study in term infants suggested an ED50 of 3.4 mg kg^−1^ in infants > 37 weeks gestational age [13]. One study found that term neonates receiving 6 mg kg^−1^ of sodium thiopental before intubation maintained vital signs closer to baseline than those given saline [14]. In our study, 40.4% of respondents reported using sodium thiopental during induction, compared to only 15.7% in a previous study [6]. This difference could be due to differences in the availability of sodium thiopental between countries. The doses for sodium thiopental herein reported (median 3 mg kg^−1^) were similar to the ED50 found for infants > 37 weeks, with some respondents administering up to 7 mg kg^−1^ for induction.

Similarly, ketamine is often used for anesthesia and analgesia in neonates and infants, valued for providing stable hemodynamics but pharmacologic studies in preterm or even term infants are lacking. In our study, 47.4% of respondents used ketamine during induction, which is substantially higher than the 8.1% reported in a previous study [6]. The median ketamine dose administered was 2 mg kg^−1^, with a range of 0.5 to 4 mg kg^−1^.

In our study, propofol was the third most used anesthetic agent for induction while it was the predominantly used agent in a previous study [6]. The optimal dosing of propofol in preterm infants is not well established, but a recent review suggested a surprisingly high range of 3 to 5 mg kg^−1^ for induction [4]. In a study of the population pharmacokinetics of propofol using pooled data from previous studies in infants, the authors reported dramatic changes in clearance defined both by gestational age (GA) and post‐natal age [15]. This suggests that a dose‐finding study in preterm infants may be challenging in this population, and indeed an attempt was terminated early because both the sedative effect and occurrence of hypotension were unpredictable [16], which might be caused by a considerable inter‐individual variability in propofol clearance [17]. Furthermore, another study that was discontinued reported substantial hypotension in preterm infants following propofol boluses as low as 1 mg kg^−1^ (doses ranged from 1 to 5 mg kg^−1^) [18]. In summary, these findings highlight that propofol dosing in preterm infants solely based on weight may lead to over‐dosing and subsequent cardiovascular risk.

### 4.2 Maintenance of Anesthesia

For maintenance of anesthesia, we identified 13 unique combinations among respondents. Fentanyl together with sevoflurane was the most commonly used combination for maintenance of anesthesia in the Nordic countries, with ketamine often used as an additional agent. A similar strategy was reported in a previous European survey although here midazolam was the most used additional agent instead of ketamine [6]. Sevoflurane is the most widely used volatile anesthetic globally. In preterm infants, MAC is influenced by gestational and postmenstrual age, with one study showing that preterm infants born at a gestational age of 30 weeks had a MAC of 2.5% at a postmenstrual age of 39 weeks and a MAC of 3% at a postmenstrual age of 54 weeks [19]. Similar findings for isoflurane indicate lower MAC values in preterm infants born at less than 32 weeks compared to those born week 32–37 [20]. In our study, the median end‐tidal concentration of sevoflurane used was 1.5%, with a range of 0.3% to 3.5%. As mentioned above, the wide range may reflect different strategies to reduce the dose by adding high‐dose opioids or ketamine.

### 4.3 Neuromuscular Blocking Agents (NMBA)

Rocuronium and atracurium were the most commonly used neuromuscular blocking agents (NMBAs), each being used in approximately 40% of cases. There was considerable variability in the intervals for administering subsequent doses. Rocuronium is used off‐label in this population but some pharmacologic data is available. Following an induction dose of 0.5 mg kg^−1^ rocuronium in preterm infants, the mean onset of paralysis was 65.9 s (± 43.3 s), with a mean time to recovery of voluntary movements, including breathing, of 16 min (± 13 min) [21]. A study on atracurium in neonates, including low‐weight infants, found that the duration of effect varied with chronological age and body temperature. Patients with a body temperature above 36°C had a mean duration of effect of 27 min after a 0.5 mg kg^−1^ dose, while those below 36°C had an effect lasting 47.5 min, with a duration range of 17 to 70 min across all patients [22]. In summary, while there is some PD data, reliable PK data are lacking both for rocuronium and atracurium in preterm infants.

### 4.4 Ventilation

The debate about the benefits and risks of supplemental oxygen for preterm infants and neonates is on‐going since over 70 years. A meta‐analysis comparing higher (91%–95%) to lower (85%–89%) concluded that the higher risk of death and necrotizing enterocolitis with the lower target must be weighed against the lower risk of retinopathy of prematurity [23]. How this translates into the intra‐operative setting needs further study. Thus, European consensus guidelines recommended targeting saturations between 90% and 94% [24] in the NICU but no evidence‐based recommendations exist specifically for the perioperative period. In our study, the median lowest and highest acceptable saturations were 90% and 96%, respectively, aligning with European NICU guidelines. However, a broad range was noted for both acceptable lowest and highest saturations.

A recent review endorses permissive hypercapnia with a pCO_2_ range of 5–7 kPa as the safest approach, consistent with current European consensus guidelines [24, 25]. In contrast, hypocapnia (pCO_2_ < 4.7 kPa) increases the risk of necrotizing enterocolitis, bronchopulmonary dysplasia, retinopathy of prematurity, and intraventricular hemorrhage, and should be avoided [25]. Hypocapnia can occur due to overventilation, and volume‐targeted ventilation modes have been shown to reduce mortality and morbidity in preterm infants compared to pressure‐limited ventilation in the NICU [26]. In our study, a majority of anesthesiologists reported using pressure‐controlled ventilation, which aligns with findings from the APRICOT study [27]. The median lowest and highest EtCO_2_ levels were 4.5 and 6.5 kPa, respectively. Tight control of ventilation and CO_2_ levels in perioperative settings may help mitigate risks associated with pressure‐controlled ventilation and overventilation. Additionally, European consensus guidelines recommend a tidal volume of 5–7 mL kg^−1^. The median tidal volume reported by anesthesiologists in our study conformed to this recommendation, although the reported range of 4–12 mL kg^−1^ is a concern and indicates the need for further study.

### 4.5 Blood Pressure Management

In 1992, a joint working group established that measuring blood pressure in preterm infants and neonates is crucial for recognizing hypotension and addressing underlying causes. They recommended a mean arterial pressure (MAP) equivalent to the gestational age in weeks as a minimum value until further research provided normal blood pressure ranges for awake preterm infants [28]. This threshold is often applied by neonatologists in European NICUs [29]. Since then, various studies have explored normal blood pressure ranges in preterm infants, summarized in a systematic review [30]. However, variations in gestational age, timing of measurements, and methods complicate the application of these findings in clinical practice. A recent retrospective cohort study provided non‐invasive blood pressure reference values for awake preterm infants during the first week of life (gestational age range 24–41 weeks) and indicated a steep rise in blood pressure during the first 24 h, followed by a gradual increase. Notably, over 97.7% of awake preterm infants had a MAP higher than their gestational age from day 1 to 2 after birth [31]. Although these reference values are not directly applicable to the intra‐operative setting with general anesthesia, it gives an indication of the normal blood pressure range in awake preterm infants. In our study, most respondents adhered to the recommendation established over 30 years ago, considering a MAP lower than gestational age as the threshold for intervention. However, when asked for a specific lowest acceptable MAP value for a preterm infant at 27 weeks gestational age, the median was 30 mmHg, while 14% of respondents would accept a MAP lower than 27 mmHg. A prospective study of the effects on patient outcome of various levels of intra‐operative hypotension in preterm infants is warranted.

### 4.6 Vasopressor and Inotropes

Most respondents (70.1%) initiate vasopressor treatment based on MAP values. Historically, pediatricians and neonatologists favored dopamine and dobutamine, which were predominantly used as first‐ and second‐line treatments in preterm infants in the NICU [29]. Recently, anesthesiologists reported that the most commonly used vasopressors to counteract anesthesia‐induced vasodilation in preterm infants are noradrenaline, followed by dopamine and adrenaline, which is consistent with our results [6]. A 2024 review summarized current knowledge on inotropes and vasopressors in preterm infants [32]. While attempts were made to compare cardiovascular agents, most studies lacked methodological comparability, leading to unclear recommendations. Again, pharmacological and outcome studies of vasopressors in pre‐term infants under general anesthesia are needed.

### 4.7 Transfusion Thresholds

Preterm infants have lower hemoglobin levels at birth compared to term infants, with a more pronounced and earlier physiological decline in hemoglobin levels. Two studies examining high versus low hemoglobin threshold values for transfusion in preterm infants found no significant difference [33, 34], leading to a clinical practice guideline for red blood cell transfusion thresholds [35]. According to the guideline, the thresholds for preterm infants requiring respiratory support are 11 g dL^−1^ for postnatal week 1, 10 g dL^−1^ for week 2, and 9 g dL^−1^ for postnatal weeks ≥ 3. For those on minimal or no respiratory support (defined as nasal cannula flow rate < 1 L min^−1^), the thresholds are 10 g dL^−1^, 8.5 g dL^−1^, and 7 g dL^−1^, respectively.

While the current study did not address platelet transfusion thresholds, a randomized clinical trial found that a high platelet count threshold (50 × 10^9^/L) was associated with higher rates of death and major bleeding compared to a low threshold (25 × 10^9^/L) at day 28 [36]. Furthermore, the high threshold group experienced increased mortality rates and neurodevelopmental impairment at a corrected age of 2 years [37]. These thresholds are widely adopted in NICUs across Europe [38].

Although not directly applicable, these studies and guidelines may assist anesthesiologists in perioperative decision‐making for preterm infants. In our study, the median perioperative blood transfusion threshold for a 3‐week‐old preterm infant was 10 g dL^−1^, which is similar to previous reports [6] and slightly higher than the guideline recommendation of 9 g dL^−1^. Notably, 10 respondents (17.5%) indicated lower transfusion thresholds than 9 g dL^−1^, with two respondents citing thresholds of 6 and 5 g dL^−1^, respectively.

### 4.8 Fluid Resuscitation

Historically, albumin has been a commonly utilized perioperative volume expander in preterm infants [39]. Only two NICU‐based studies have compared 5% albumin with crystalloids, specifically 0.9% saline. In the first study, the albumin group required more additional fluid and experienced greater weight gain at 24 and 48 h [40]. The second study reported a reduced need for vasopressors compared to 0.9% saline [41]. Nevertheless, a Cochrane meta‐analysis concluded that the significance of these findings is unclear, with no other clinical benefits noted for 5% albumin over 0.9% saline [42]. A recent guideline does not support the use of albumin as a volume expander in preterm infants, although specific recommendations for perioperative settings are not provided [43]. Furthermore, there is no evidence to justify the use of plasma as a volume expander in cases of hypotension [44], and an observational study shows that it is rarely administered in the NICU for this indication [45]. These findings present challenges for anesthesiologists in translating recommendations into clinical practice during anesthesia. Interestingly, most respondents identified 5% albumin as the first‐choice fluid for resuscitation in hypotensive, non‐bleeding preterm infants, followed by Ringer's acetate and 0.9% saline.

## 5 Limitations

The strength of this study lies in providing real‐world dosing data for induction and maintenance anesthetic agents in preterm infants, as used by responders in clinical practice, along with thresholds for specific interventions. However, several limitations must be considered. Firstly, reporting bias is possible in a survey study. Participants may exaggerate or moderate their responses compared with actual clinical practice. However, we believe the multidimensional approach including a fictive case could reduce this type of bias. Secondly, we could not report the overall response rate for the 18 hospitals included; the response rate for the nine hospitals that disclosed email distributions was 30.4%, leading to potential selection bias. However, the variability observed among responders in our study aligns with findings from previous similar studies in both preterm infants and children [6, 46], which would likely remain unchanged even with more responses. This heterogeneity probably reflects true variation rather than a bias introduced by the response rate. Additionally, the survey was conducted across Nordic countries, which may limit the generalizability of the results to other regions. While most respondents within the same institution reported similar induction and maintenance agents, this may reflect local practices rather than a broader consensus. Response bias could affect our results since participation was voluntary, potentially skewing the data. These limitations highlight the need for cautious interpretation of our findings, as they may affect their reliability and generalizability.

## 6 Conclusions

We demonstrated substantial heterogeneity in anesthesia practice for preterm infants in spite of targeting a geographically limited and socio‐economically homogenous cohort. Furthermore, in contrast to the many studies in neonatology and the increasing evidence available for managing preterm infants in the NICU, our review of the literature found a troubling lack of research focusing on this same group of infants during the perioperative period. Currently, anesthesia management in the OR appears to rely primarily on individual clinicians' experience and hospital‐dependent local consensus. The demonstrated variability in practice underscores the urgent need for more research to understand the pharmacology of the most common anesthetic agents in the OR as well as optimal strategies to support hemodynamics and ventilation. Evidence‐based guidelines are currently lacking.

## Author Contributions

**Jaap van der Heijden:** conceptualization, methodology, software, formal analysis, investigation, writing – original draft, visualization. **Peter Frykholm:** conceptualization, writing – review and editing, supervision.

## Funding

The authors have nothing to report.

## Conflicts of Interest

The authors declare no conflicts of interest.

## Supporting information


**Table S1:** Blood pressure target as threshold for intervention.
**Table S2:** Maximal accepted reduction from NIRS baseline before increasing FiO_2_.
**Figure S1:** Heatmap showing the frequency of combinations of agents per hospital for induction of anesthesia of preterm infants. (F)entanyl, (R)emifentanil, (K)etamine, (P)ropofol, (S)evoflurane, sodium (T)hiopental, (L)orazepam, (M)idazolam.
**Figure S2:** Heatmap showing the frequency of combinations of agents per hospital for maintenance of anesthesia of preterm infants. (F)entanyl, (R)emifentanil, (K)etamine, (P)ropofol, (S)evoflurane, (I)soflurane, sodium (T)hiopental, (L)orazepam, (M)idazolam.

**Data S1:** Supplemental Survey.

## Data Availability

The data supporting the findings of this study are available upon reasonable request from the corresponding author.
