# Supplementary material for: Anesthesia Practices for Preterm Infants: A Survey in the Nordic Countries and Review of the Literature
Source: Acta Anaesthesiol Scand. 2026 Feb 12;70(3):e70186. doi: 10.1111/aas.70186 (PMC12895386; doi:10.1111/aas.70186)
Supplement: Supplementary file 1 — Table S1: Blood pressure target as threshold for intervention. Table S2: Maximal accepted reduction from NIRS baseline before increasing FiO2. Figure S1: Heatmap showing the frequency of combinations of agents per hospital for induction of anesthesia of preterm infants. (F)entanyl, (R)emifentanil, (K)etamine, (P)ropofol, (S)evoflurane, sodium (T)hiopental, (L)orazepam, (M)idazolam. Figure S2: Heatmap showing the frequency of combinations of agents per hospital for maintenance of anesthesia of preterm infants. (F)entanyl, (R)emifentanil, (K)etamine, (P)ropofol, (S)evoflurane, (I)soflurane, sodium (T)hiopental, (L)orazepam, (M)idazolam. [file AAS-70-0-s002.docx]

| **Supplemental Table S1. Blood pressure target as threshold for intervention.** | | | |
| --- | --- | --- | --- |
|  | count (n) | % of total | Thresholds |
| MAP = GA | 49 | 86.0 | MAP < GA |
| Capillary refill time | 1 | 1.8 | > 3 seconds |
| Fixed value | 2 | 3.5 | <30 mmHg; <35 mmHg |
| Other | 1 | 1.8 | “Lactate, NIRS, SvO2” |
|  | 1 | 1.8 | “MAP around GA” |
|  | 1 | 1.8 | “Gestational age + 3” |
|  | 1 | 1.8 | “MAP > GA, in combination with NIRS” |
|  | 1 | 1.8 | “Depending on the status” |

| **Supplemental Table S2. Maximal accepted reduction from NIRS baseline before increasing FiO2** | | |
| --- | --- | --- |
|  | count (n) | % |
| No fixed | 15 | 65.2 |
| Max 5% | 0 | 0 |
| Max 10% | 3 | 13.0 |
| Max 15% | 1 | 4.3 |
| Max 20% | 4 | 17.4 |


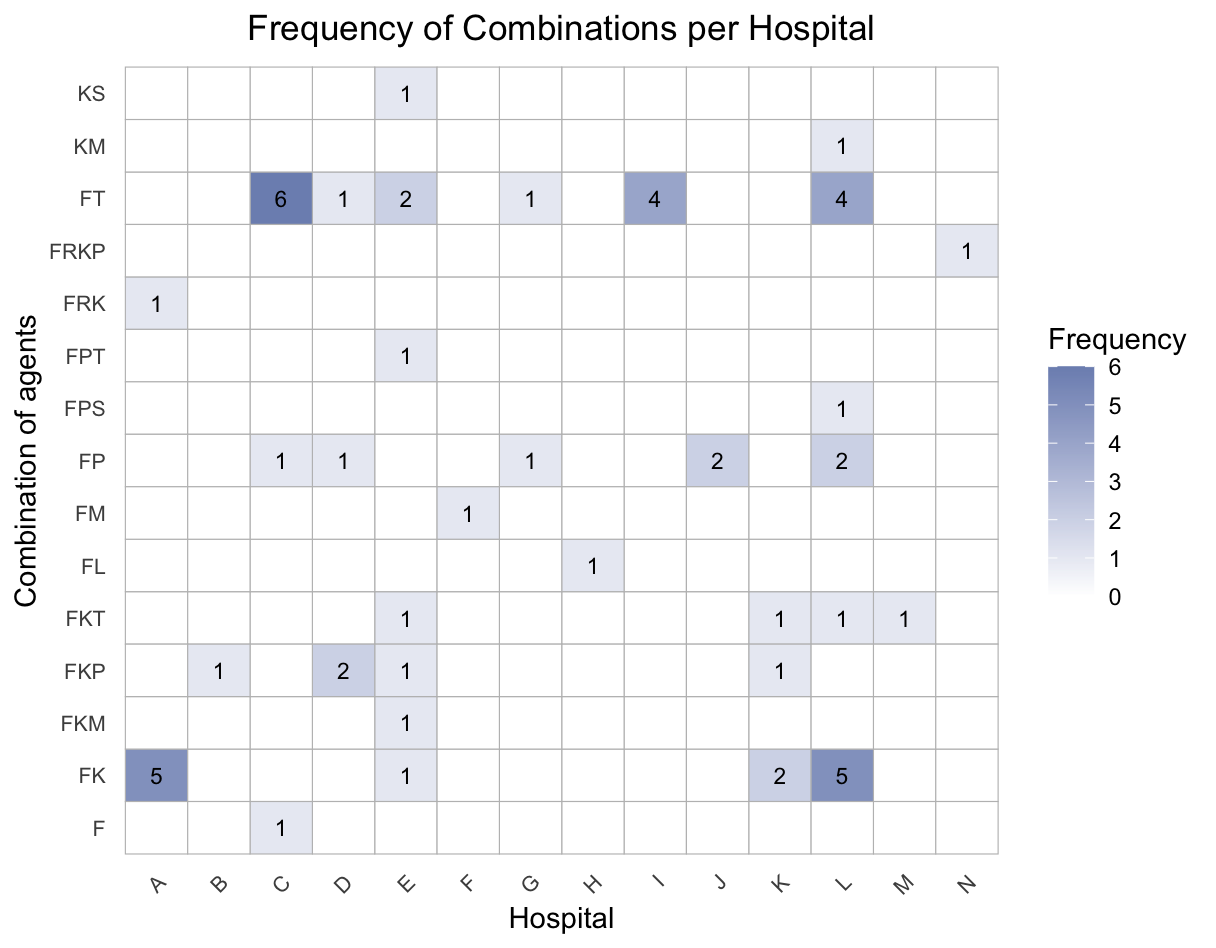


**Supplemental Figure S1. Heatmap showing the frequency of combinations of agents per hospital for induction of anesthesia of preterm infants.**

**(F)entanyl, (R)emifentanil, (K)etamine, (P)ropofol, (S)evoflurane, sodium (T)hiopental, (L)orazepam, (M)idazolam**


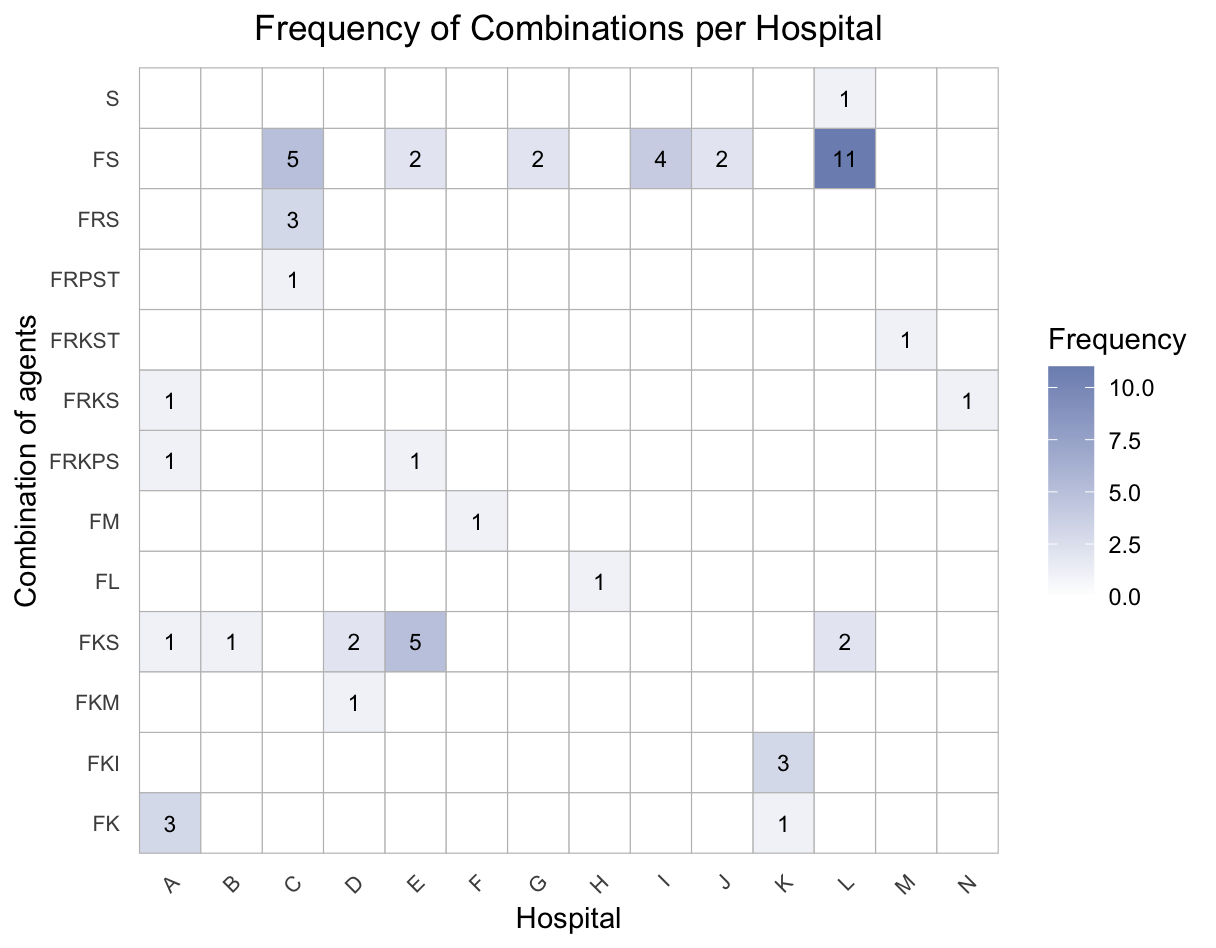


**Supplemental Figure S2. Heatmap showing the frequency of combinations of agents per hospital for maintenance of anesthesia of preterm infants.**

**(F)entanyl, (R)emifentanil, (K)etamine, (P)ropofol, (S)evoflurane, (I)soflurane, sodium (T)hiopental, (L)orazepam, (M)idazolam**
