# Supplementary material for: Anesthesia Practices for Preterm Infants: A Survey in the Nordic Countries and Review of the Literature
Source: Acta Anaesthesiol Scand. 2026 Feb 12;70(3):e70186. doi: 10.1111/aas.70186 (PMC12895386; doi:10.1111/aas.70186)
Supplement: Supplementary file 2 — Data S1: Supplemental Survey. [file AAS-70-0-s001.pdf]

Survey

Please complete the survey below.

Thank you!

---

1 Age
(number only) ________________________

2 Gender
- ◯ female
- ◯ male
- ◯ prefer not to say
- ◯ other

3 Hospital currently working
- ◯ Aalborg
- ◯ Aarhus
- ◯ Bergen
- ◯ Copenhagen
- ◯ Gothenburg
- ◯ Helsinki
- ◯ Kuopio
- ◯ Lund
- ◯ Odense
- ◯ Oslo
- ◯ Oulu
- ◯ Reykjavik
- ◯ Stockholm
- ◯ Tampere
- ◯ Tromsø
- ◯ Trondheim
- ◯ Turku
- ◯ Uppsala

4 Years of experience after specialist training
(number only) ________________________

5 Years of experience in paediatric
anaesthesia/intensive care (number only) ________________________

6 Number of anaesthesia provided on preterm infants last
12 months ________________________

Preterm defined as gestational age < 37weeks
(number only)

7 Anaesthesia on preterm infants at daytime is in
general managed by me
- ◯ as a single specialist
- ◯ together with at least one more specialist

8 Anaesthesia on preterm infants during after office
hours is in general managed by me
- ◯ as a single specialist
- ◯ together with at least one more specialist

9 How often is the Neonatal Intensive Care Unit (NICU)
specialist in general participating during an
operation (in %)

never ______________________________ always

(Place a mark on the scale above)

10 Are you authorised to independently manage general anaesthesia for preterm infants at your clinic.

◯ Yes
◯ No

When selecting 'No' the survey will stop as the following questions should be answered ONLY by those who are authorised to manage anaesthesia independently.

ALL of the following questions should be answered in context with the following case

S - acute necrotizing enterocolitis (NEC)
B - premature, born week 24, gestational age week 27, mild BPD (bronchopulmonary dysplasia)
A
a - normal facial anatomy, previously intubated and extubated without problems.
b - intubated, FiO2 45%, mechanically ventilated with peak pressure 24, PEEP 7, transcutaneous CO2 6.
c - adequate blood pressure and heart rate without vasopressor support, electrolytes within normal range, peripherally warm and perfused with capillary refill < 2 seconds, Hb 14 g/dL
d - no signs of syndrome or neurological defects, signs of bowel obstruction

R - acute laparotomy

11 Medication used for maintenance of general anaesthesia (multiple answers possible)

☐ fentanyl
☐ alfentanil
☐ remifentanil
☐ sufentanil
☐ ketamine
☐ propofol
☐ sevoflurane
☐ isoflurane
☐ other

Fentanyl selected, specify what intermittent bolus dose in general is given (in mcg/kg) ______________________________

Alfentanil selected, specify what intermittent bolus dose in general is given (in mcg/kg) ______________________________

Remifentanil selected, specify what infusion rate in general is given (in mcg/kg/min) ______________________________

Sufentanil selected, specify what intermittent bolus dose in general is given (in mcg/kg) ______________________________

Ketamine selected, specify what intermittent BOLUS dose in general is given (in mg/kg) ______________________________

OR

Ketamine selected, specify what INFUSION rate in general is given (in mg/kg/h) ______________________________

Propofol selected, specify what infusion rate in general is given (in mg/kg/h) __________________________________

Sevoflurane selected, specify what end-tidal sevoflurane in general is used (in %) __________________________________

Isoflurane selected, specify what end-tidal isoflurane in general is used (in %) __________________________________

Other selected, please specify which medication __________________________________

Other selected, please specify which intermittent bolus dose or infusion rate in general is given (intermittent bolus in mcg or mg per kg) (infusions in mcg or mg per kg per minute or hour) __________________________________

12 Which neuromuscular blockage agent (NMBA) is in general given peri-operative

- ◯ rocuronium
- ◯ atracurium
- ◯ cis-atracurium
- ◯ mivacurium
- ◯ NMBA rarely given
- ◯ other

Rocuronium selected, specify what bolus dose in general is given (mg/kg) __________________________________

Atracurium selected, specify what bolus dose in general is given (mg/kg) __________________________________

Cis-atracurium selected, specify what bolus dose in general is given (mg/kg) __________________________________

Mivacurium selected, specify what bolus dose in general is given (mg/kg) __________________________________

Other selected, please specify which medication __________________________________

Other selected, specify what bolus dose in general is given (mg/kg) __________________________________

13 What is in general the interval (in minutes) for administering NMBA during general anaesthesia of a preterm infant __________________________________

14 Which monitoring is in general used during general anaesthesia of a preterm infant (multiple answers possible)

- ☐ SpO2
- ☐ non-invasive blood pressure
- ☐ invasive blood pressure
- ☐ NIRS (Near-infrared spectroscopy)
- ☐ sidestream CO2
- ☐ mainstream CO2
- ☐ microstream CO2
- ☐ transcutaneous CO2
- ☐ other

SpO2 selected, how many pulse oximeters are used
- ◯ 1
- ◯ 2
- ◯ >2

Invasive blood pressure selected, which of the following is true
- ◯ these patients normally come with an umbilical artery catheter from NICU
- ◯ arterial line is placed by the neonatologist
- ◯ arterial line is placed by the anaesthetist

NIRS selected, what reduction from baseline is accepted before increasing FiO2
- ◯ maximum 5% decrease
- ◯ maximum 10% decrease
- ◯ maximum 15% decrease
- ◯ maximum 20% decrease
- ◯ maximum 30% decrease
- ◯ no fixed %

Other selected, please specify

____

15 Which continuous CO2 monitoring do you consider to be most reliable in preterm infants?
- ◯ sidestream CO2
- ◯ mainstream CO2
- ◯ microstream CO2
- ◯ transcutaneous CO2
- ◯ other

Other selected, please specify

____

16 What is your target of (Et)CO2 during general anaesthesia for preterm infants (number only [in kPa])

LOWEST target range:

To convert from mmHg to kPa: divide mmHg value by 7.5

____

17 What is your target of (Et)CO2 during general anaesthesia for preterm infants (number only [in kPa])

HIGHEST target range:

To convert from mmHg to kPa: divide mmHg value by 7.5

____

18 Mode of ventilation used in general
- ◯ pressure controlled
- ◯ volume controlled
- ◯ pressure-controlled volume guaranteed (also known as pressure-regulated volume control)
- ◯ other

Other selected, please specify

____

19 Tidal volume used in general (in ml/kg)

____

20 Is tidal volume based on inspiratory or expiratory values
- ◯ inspiratory values
- ◯ expiratory values

21 What is your target range saturation during general anaesthesia for preterm infants (in %).

LOWEST target: ______________________

22 What is your target range saturation during general anaesthesia for preterm infants (in %).

HIGHEST target: ______________________

23 Blood pressure target as threshold for intervention is based on

- ◯ systolic pressure
- ◯ diastolic pressure
- ◯ mean arterial pressure (MAP)
- ◯ no blood pressure target used
- ◯ blood pressure not regular taken

24 Blood pressure target as threshold for intervention is dependent on

- ◯ gestational age in weeks (MAP ≥ gestational age)
- ◯ fixed value for all preterm infants
- ◯ capillary refill time (CRT)
- ◯ other

Fixed MAP selected, please report value (mmHg) ______________________

Capillary refill time selected, please report threshold value (CRT more then X seconds) ______________________

Other selected, please specify ______________________

25 What is your lowest acceptable MAP for this patient? ______________________

26 What strategy is used for vasopressor support in preterm infants

- ◯ vasopressor almost never given
- ◯ strictly depending on blood pressure target
- ◯ routinely used from the start
- ◯ other

Other selected, please specify ______________________

27 If the patient is euvolemic and hypotensive, which vasopressor is the first choice to give as infusion in preterm patients?

- ◯ adrenaline
- ◯ dobutamine
- ◯ dopamine
- ◯ noradrenaline
- ◯ phenylephrine
- ◯ other

Other selected, please specify ______________________

28 If the patient is euvolemic and hypotensive, is in general calcium gluconate given as an inotrope?

- ◯ Yes
- ◯ No

29 The patient is hypotensive based on hypovolemia (non bleeding, pre-op Hb 14 g/dL)
What is the first choice of fluid resuscitation?

- ◯ albumin 5% = 50g/L
- ◯ albumin 20% = 200g/L
- ◯ normal saline 0.9% = 9mg/ml
- ◯ ringer-acetate
- ◯ other

Albumin 5% selected, please specify amount (ml/kg) ______________________

Albumin 20% selected, please specify amount (ml/kg) ______________________

Normal saline (0.9%) selected, please specify amount (ml/kg) ______________________

Ringer-acetate selected, please specify amount (ml/kg) ______________________

Other selected, please specify which fluid ______________________

Other selected, please specify what amount (ml/kg) ______________________

---

30 Which transfusion threshold would you use for this case ______________________

(please note that values should be reported as g/dL)

(as a reference: Hb 100 g/L = 10 g/dL)

The following question is in case this patient is not yet intubated on arrival in the OR.
Which of the following medications are used for induction of general anaesthesia?

---

31 Medication used for induction of anaesthesia (multiple answers possible)

- ☐ fentanyl
- ☐ alfentanil
- ☐ remifentanil
- ☐ sufentanil
- ☐ ketamine
- ☐ propofol
- ☐ sodium thiopental
- ☐ rocuronium
- ☐ atracurium
- ☐ cis-atracurium
- ☐ mivacurium
- ☐ succinylcholine
- ☐ atropine
- ☐ glycopyrrolate
- ☐ other

Fentanyl selected, please specify dose (in mcg/kg) ______________________

Alfentanil selected, please specify dose (in mcg/kg) ______________________

Remifentanil selected, please specify dose (in mcg/kg) ______________________

Sufentanil selected, please specify dose (in mcg/kg) __________________________________

Ketamine selected, please specify dose (in mg/kg) __________________________________

Propofol selected, please specify dose (in mg/kg) __________________________________

Sodium thiopental selected, please specify dose (in mg/kg) __________________________________

Rocuronium selected, please specify dose (in mg/kg) __________________________________

Atracurium selected, please specify dose (in mg/kg) __________________________________

Cis-atracurium selected, please specify dose (in mg/kg) __________________________________

Mivacurium selected, please specify dose (in mg/kg) __________________________________

Succinylcholine selected, please specify dose (in mg/kg) __________________________________

Atropine selected, please specify dose (in mcg/kg) __________________________________

Glycopyrrolate selected, please specify dose (in mcg/kg) __________________________________

Other selected, please specify which medication __________________________________

Other selected, please specify dose (report mcg or mg per kg) __________________________________

32 If needed please clarify some of the answers in the textbox (add question number) ____
